# Evaluation of the effect of lidocaine epidural injection on immunological indices in dogs under total intravenous anesthesia submitted to ovariohysterectomy

**DOI:** 10.1371/journal.pone.0253731

**Published:** 2021-06-28

**Authors:** Hadi Imani Rastabi, Mohammad Khosravi, Reza Avizeh, Maryam Moslemi

**Affiliations:** 1 Faculty of Veterinary Medicine, Department of Clinical Sciences, Shahid Chamran University of Ahvaz, Ahvaz, Iran; 2 Faculty of Veterinary Medicine, Department of Pathobiology, Shahid Chamran University of Ahvaz, Ahvaz, Iran; PLOS ONE, UNITED KINGDOM

## Abstract

This study aimed to evaluate the effects of epidural anaesthesia with lidocaine in combination with general anaesthesia with propofol on some immunologic indices in dogs undergoing ovariohysterectomy. Twelve adult dogs were anesthetized with propofol (induction: 7 mg/kg; maintenance: 0.4 mg/kg/min) and were then allocated into either groups of epidural saline (control) or epidural lidocaine (4 mg/kg; treatment). All the included animals underwent ovariohysterectomy operation. The immune responses, hematologic parameters and cortisol levels were assessed in the predetermined intervals. Evaluation of the innate immunity revealed higher significant levels in the bactericidal, lysozyme and myeloperoxidase activities at 4 hours after surgery in the treatment. In the humoral immunity, the total immunoglobulin level was significantly higher in the treatment. In the assessment of cellular immunity, higher significant values were detected in the delayed skin sensitivity to phytohemagglutinine injection after 48 and 72 hours in the treatment. Moreover, higher significant levels were observed in the number and percentage of lymphocytes as well as an increase in the percentage of monocytes in the treatment at 4 hours after the operation. Notably, the cortisol hormone in the treatment was lower than control at 4 hours of the surgery. In conclusion, epidural anaesthesia with lidocaine when added to general anaesthesia with propofol attenuated the suppression of the innate and cellular immune responses produced by anaesthesia and surgery in the dogs.

## Introduction

Surgery is accompanied with various degrees of immunosuppression. Accordingly, this is of great importance because immunosuppression in the perioperative period can affect the long-term outcome and also provokes some postoperative adverse effects including a delay in wound healing and occurrence of infection [1]. Furthermore, the immune disturbances have recently received more concerns in oncology procedures, since it speeds-up the development of the remained cancer cells as well as increasing the chance subsequent metastasis [2]. Operation, by itself, is associated with the trauma-induced stress which subsequently leads to the activation of hypothalamic-pituitary–adrenal axis and secretion of catecholamines and glucocorticoids which have direct immunosuppressive effects [3]. On the other hand, it has been shown that most of the anaesthetic agents impair the activity of immune system at cellular level [4–6]. Moreover, anaesthesia and analgesia can also modulate surgical stress and affect the immune response [7].

Although both volatile and injectable anesthetics suppress the immune functions, it is generally accepted that the immunosuppressive impacts of total intravenous anesthesia (TIVA) are lower than that of the inhalation anaesthesia [8]. Propofol, as an injectable anesthetic provides rapid induction, short duration, fast elimination and smooth recovery; is mostly used for TIVA in veterinary medicine. In this regard, some studies have suggested that propofol might have some protective properties on immunological functions in human beings and dogs [9,10].

Local anaesthesia, produced by local anesthetics, can be employed before, during and after surgery to provide perioperative anaesthesia and/or analgesia. Correspondingly, several studies have shown the superiority of local anaesthesia over general anaesthesia on immunity responses during performing painful procedures [11–13]. Local anaesthesia might be used alone or in combination with general anaesthesia. It has been indicated that the combination of local and general anaesthesia attenuates postoperative immune suppression and also reduces the neuroendocrine response to surgery [14]. The positive effect of local anaesthesia when added to general anaesthesia on the reduction of immunosuppression; and, consequently possible metastasis and tumor recurrence in patients with cancer are of interest, which have been documented by a number of investigations [15–19].

To the best of the authors’ knowledge, no study has evaluated the effect of combination of general and local anaesthesia on the immunological responses in veterinary medicine. Thus, the purpose of the current study was to compare the impacts of general anaesthesia (TIVA with propofol) and the combination of general (TIVA with propofol) and epidural anaesthesia (with lidocaine) on the immunological indices indicating innate, humoral, and cellular immunities in dogs undergoing ovariohysterectomy (OHE). In this regard, it was expected that the addition of epidural anaesthesia to general anaesthesia promotes the immunological responses.

## Material and methods

### Animals

The present study was an experimental double-blinded investigation. At first, a total of 16 dogs were enrolled. The animals belonged to a private shelter designed and built for housing vendor and stray dogs. The dogs were transferred to the Hospital of Veterinary Medicine at least two weeks prior to the study and maintained in separate cages (1 m × 1.5 m × 1.2 m) until two weeks after the completion of the experiments. The room temperature was about 30–35°C. It was lit at 6 pm to 7:30 am. Dogs were kept indoors all day without outdoor exercise but they were allowed to be free out of the cage for about 15–20 minutes at the time of feeding. The animals’ health status was determined by performing complete physical examination, cell blood count and total protein measurements. A vaginal smear was taken which was microscopically evaluated to confirm that the animals were in anestrus phase. Excluding criteria were pregnancy, being out of anestrus, obvious inflammatory conditions (e.g. local or systemic disease, skin wounds or painful responses during palpation), aggressive behaviour and any hematologic crises in CBC. According to the above-mentioned exclusion criteria, of the 16 dogs that were initially enrolled, 12 adult mongrel bitches weighing 16.1 ± 1.6 kg and aged 1.5–2.5 years old were included in the present study. The excluded dogs were returned to their origin place (i.e. private shelter). During the study, animals were fed (commercial dog food plus head and neck of chicken) twice a day (9:00 am and 5:00 pm) with free access to water. Before performing each experiment, 12 hours of food limitation and 2 hours of water avoidance were applied. After the completion of the study, the dogs were returned to the private shelter. During the study and follow-up of at least 4 weeks after the end of the experiments, none of the dogs showed any adverse effect resulting in sickness or death. The average time, which dogs were under care, was approximately 2 months. The ethics committee of Shahid Chamran University of Ahvaz approved all the procedures of the current investigation (EE/96.24.3.58888/SCU.ac.ir).

### Study design

The dogs were randomly assigned to either receive treatments (six dog in each group) of 1- group PS: TIVA with propofol (loading dose: 7 mg/kg; constant rate infusion: 0.4 mg/kg/min) (Propofol Lipuro 10 mg/ml, Melsungen, Germany) and epidural application of normal saline (0.22 mL/kg) or 2- group PL: TIVA with propofol (loading dose: 7 mg/kg; constant rate infusion: 0.4 mg/kg/min) and epidural anaesthesia with lidocaine (4 mg/kg) (Lignodic, lidocaine hydrochloride 20 mg/ml, Caspian Tamin, Rasht, Iran). The final volume of epidural solutions was adjusted to 0.22 mL/kg using 0.9% normal saline. The randomization was performed using an Internet website designed for the research purposes (www.randomizer.com).

The innate immune status and humoral and cell-mediated immune responses, hematological changes and the levels of cortisol were assessed, determined and recorded at baseline and at also at the predetermined intervals. For humoral immune evaluation, 5 days prior the initiation of the experiments, 1 mL of antigens mixture solution containing SRBC (10%), BSA (1 mg/kg) and killed *Salmonella typhimurium* in sterilized normal saline were intramuscularly (IM) injected. Moreover, to assess cellular immune activity, 2 hours prior to the experiment, a 5 × 5 cm skin of the both sides of neck was clipped and cleaned. Afterwards, 0.1 mL phosphate buffered saline and 0.1 mL phytohemagglutinin (0.5 mg/mL) were intradermally injected into the right and left sides, respectively. The area of injection was marked. All the injections were performed by an investigator (M.K).

### Anaesthesia

After transferring to the place of the study, the dogs received acepromazine (0.025 mg/kg) (Neurotranq, acepromazine maleate 10 mg/ml, Alfasan, Woerden-Holland) and morphine (0.25 mg/kg) (Morphine sulfate 10 mg/ml, Darou Pakhsh, Iran) as sedation IM into the bulk of the right hamstring muscles. Forty minutes later, the left and right antebrachial areas were clipped and aseptically prepared. Subsequently, the left (for saline infusion) and right cephalic (for propfol injection) veins were catheterized using a proper-sized intravenous catheter. After pre-oxygenation with oxygen 100% for 5 minutes via a facemask positioned on the muzzle, anaesthesia was induced and then maintained with propofol. Then, the trachea was intubated with a suitable cuffed tracheal tube. The dogs were maintained breathing oxygen 100% (100 mL/kg/min) via the tracheal tube up to the end of the anaesthesia. Normal saline was infused at the rate of 10 mL/kg/h after induction until the end of the surgery. Depth of anaesthesia was assessed every 5 minutes and maintained at a suitable plan to enable performing operation using pedal and palpebral reflexes, eyeball position, jaw tone and alterations in cardiorespiratory variables to surgical stimulation. If the depth of anaesthesia was not acceptable, the dose of propofol would be adjusted 10% accordingly.

Then, the animals were positioned in sternal recumbency and thighs were cranially stretched. The skin overlying the lumbosacral space was clipped and aseptically prepared. One mL lidocaine 1% was subcutaneously injected to the site of epidural needle perforation. A 22-gauge 3.8 cm hypodermic needle was inserted into the skin and directed to the epidural space. Hanging drop test and loss of resistance against the injection were utilized for confirming correct placement of the needle in the epidural space. After negative aspiration of blood or cerebrospinal fluid, the drug was slowly injected in about 30 seconds. All the epidural injections were performed by an investigator (H.I.R).

### Surgery

Five minutes after epidural injection, the dogs were placed in dorsal recumbency. The surgical area was aseptically prepared. Ventral midline celiotomy was done using a 5-cm skin incision caudal to the umbilicus. Ovariohysterectomy was performed in routine manner described elsewhere [20]. All the operations were done by the two trained residents in veterinary surgery. At the end of surgery, the infusion of propofol was disrupted and the animal was then positioned in right lateral recumbency. All the operations were performed during 10:00 AM to 02:00 PM. At the end of the surgery, cephazoline (22 mg/kg) (Cefazolin 1g, Dana, Tabriz, Iran) and tramadol (2 mg/kg) (Tramadol 50 mg/mL, Alborz Daro, Iran) were administered IM q24h for 3 days.

### Blood sampling

Blood collections (2 mL at each session) were performed via jugular vein before and after (before sedative agents injection) antigen administrations, immediately after the induction of anaesthesia (Base), and at 4, 12, 24, 48 and 72 hours as well as at 5 and 10 days after anaesthesia. The obtained blood samples were centrifuged (3000 rpm for 10 minutes), and sera were separated which were then stored at -20°C until the time of evaluations.

### Assessments

#### Anaesthesia and surgery

Heart rate, *f*_R_, RT and non-invasive SAP, DAP, MAP and SPO_2_ were measured prior to the induction of anaesthesia (base), and at 10, 20 and 30 minutes after the induction and also at recovery time. Anesthetic monitoring device (Burtons, UK) and stethoscope were used to measure and record HR. *f*_R_ was recorded by observing and counted the chest movements. Rectal temperature was measured by a digital thermometer. Non-invasive SAP, DAP, and MAP were measured via a specific cuff (with the width of at least 40% of the circumference of the limb) closed at the metatarsus region of the left hindlimb connected to the anesthetic monitoring system.

At the end of anaesthesia, the administered amount of propofol as well as the duration of surgery and anaesthesia were recorded. The duration of surgery was defined as from the time of the incision of the skin to the last knot for the skin incision closure. The duration of anaesthesia was determined as the time of anaesthesia induction until the time of the animals’ ability to stand.

#### Innate immunity

Antibacterial effects of the animal sera were evaluated using microdilution broth method in the 96-well microplates. The antibacterial activity of each fraction was evaluated against *Staphylococcus aureus* and *Escherichia coli*. The bacterial strains were cultured on blood agar medium and then incubated overnight at 37°C. One colony from each strain was introduced into Mueller Hinton Broth at 37°C and monitored until reaching the turbidity equal to 0.5 McFarland standard. Each one of the serum samples (25 μL) was mixed with an equal volume of brain heart infusion medium in specified microplate wells. The prepared bacteria in a volume of 25 μL, were added to the wells. After reading the optical density (OD) at 600 nm using a spectrophotometer (AccuReader, Taiwan), the plates were incubated for 24 h at 37°C, and the ODs were reread under the same conditions as mentioned earlier. The results were interpreted by the calculation of the bacterial growth in the test and control wells [21].

The serum lysozyme activity was determined using the turbidimetric assay. At first, a buffer was prepared by the addition of 20 mg *Micrococcus lysodeikticus* to 100 mL acetate buffer (0.02 M, pH 5.5). Then, the buffer 150 μL was mixed with 15 μL of each serum samples in 96 microplate wells and incubated at 25°C. The OD was read at a wavelength of 450 nm after 5 minutes. The decreased OD equal to 0.001 per minute was taken as one unit of lysozyme activity [22].

Total serum myeloperoxidase activity was measured according to oxidation of 3,3′,5,5′-tetramethylbenzidine via hypochlorous acid or the peroxidation cycle of MPO to a strongly absorbed blue product. About 10 μL of serum was diluted with 90 μL of Hanks balanced salt solution without Ca2^+^ or Mg2^+^ in 96-well plates. Then 35 μL of 20 mM 3,3,5,5- tetramethylbenzidine and 5 mM H_2_O_2_ were added. The colour change reaction was stopped after 2 minutes by the addition of 35 μL of 4 M sulphuric acid. The enzyme activity was colorimetrically determined using a plate reader at a wave length of 450 nm. expressed as OD [23].

#### Humoral immunity

For the humoral immune evaluation, the amounts of total serum immunoglobulin and micro-agglutination tests including the determination of the levels of antibody against SRBC, anti-BSA and anti- *Salmonella typhimurium* antibodies, were employed. The total immunoglobulin concentration was spectrophotometrically determined by measuring the turbidity resulting from the addition of zinc sulfate to the serum. The serum samples of the dogs 15μL at 0, 4, 12, 24, 48 and 72 hours after surgery were added to 850 μL of zinc sulfate 0.7 mM (pH: 5.8) and incubated for 2 hours at room temperature. The OD of the resulted sedimentation was measured at 600 nm [24]. The humoral immune response against SRBC, BSA and *Salmonella typhimurium* were evaluated using hemagglutination, passive hemagglutination and agglutination tests respectively, in 0, 5 and 10 days after injection of the antigens according to Hay et al. [25].

#### Cellular immunity

The activity of cellular immune system was assessed via stimulus rates of T lymphocytes by the evaluation of the delayed skin sensitivity to phytohemagglutinin injection. Delayed-type hypersensitivity was assessed in all the tested dogs by measuring skin induration response to the phytohemagglutinin injection. Skin induration was measured at 0, 24 (2 hours after anaesthesia), 48 and 72 hours post-injection using a sensitive electronic digital micrometer (Sunche, China) and the response was then expressed as the percentage of the change in skin thickness [26].

#### Hematology

Hematological tests including counting the white blood cells (lymphocytes, monocytes, granulocytes and lymphocyte granulocytes), red blood cells, hematocrit and the amount of platelets were performed using a calibrated cell counter (BC-2800 Vet, China).

#### Serum cortisol hormone

The level of cortisol was measured at 0, 4, 12, 24, 48 and 72 hours after surgery using the cortisol ELISA diagnostic kit (Ideal Tashkhis Atieh, Tehran, Iran) by an ELISA reader (AccuMate Reader, Taiwan).

### Statistics

Statistical analyses were carried out using SPSS version 22 (SPSS, IBM Corporation, USA). A Kolmogorov-Smirnov test was utilized for the evaluation of normality. For the normally distributed data, an Independent Sample t-test and a repeated measure for ANOVA with Bonferroni as the post hoc test were used for the comparisons between and within the groups, respectively. A Mann-Whitney U and Cochran’s tests were employed for the comparing the non-parametric data. Mean (SD) and median (range) were used to express the parametric and non-parametric values, respectively. The statistical significant level was *p* < 0.05. The sample size of six dogs per group was determined using a repeated measure for ANOVA with type I error of 5%, power of 80% and an effect size of 0.3 based on a pilot study in three dogs received epidural lidocaine and evaluated for immunological indices and cortisol level.

## Results

All the dogs tolerated the anaesthesia and surgery procedures well and no complication was observed during and after the operation. No dog was excluded or euthanized during and after the completion of the experiment. The weights of the animals were 15.38 ± 1.73 and 16.77 ± 1.30 kg in PS and PL groups, respectively, with no significant difference between them (*p* = 0.45). Also, no significant differences were detected between the groups in terms of the duration of anaesthesia (69.33 ± 7.66 minutes in PS and 67.67 ± 5.13 minutes in PL; *p* = 0.60), the duration of surgery (32.83 ± 4.24 and 30.67 ± 5.79 minutes in PS and PL, respectively; *p* = 0.52) and the amount of the propofol consumption (42.47 ± 3.69 mL for PL and 38.28 ± 5.53 mL for PS; *p* = 0.23).

The data related to the physiologic parameters are summarized in Table 1. HR showed no significant difference between the two groups (*p* > 0.05); however, some gradually significant increases were seen over time in PS and PL (*p* < 0.05). Similarly, SAP, DAP, and MAP were not significantly different between groups (*p* > 0.05), and significant higher values were detected at several time points compared to the baseline values (*p* < 0.05). *f*_R_ was significantly higher at 30 minutes after the initiation and at the end of surgery in PS compared to PL (*p* < 0.05). *f*_R_ was also lower in those time points compared to baseline in PL (*p* < 0.05). No significant changes were observed in RT and SPO_2_ between and within groups (*p* > 0.05).

**Table 1:** Changes in physiologic variables.

Variable	Group	Base	Initiation of surgery	10 min	20 min	30 min	End of Surgery
HR (beats/min)	PS	68 ± 19	92 ± 23 † ‡	101 ± 21 † ‡	103 ± 14	105 ± 16	145 ± 17
	PL	69 ± 23	95 ± 19 † ‡	101 ± 13 † ‡	101 ± 7 † ‡	106 ± 5 † ‡	133 ± 18 † ‡
SAP (mmHg)	PS	99 ± 13	108 ± 20	120 ± 31 † ‡	127 ± 14 † ‡	122 ± 18 † ‡	121 ± 20 † ‡
	PL	96 ± 2	108 ± 19	124 ± 14 † ‡	131 ± 10 † ‡	125 ± 13 † ‡	123 ± 10 † ‡
DAP (mmHg)	PS	59 ± 17	73 ± 16	82 ± 11 † ‡	86 ± 13 † ‡	85 ± 10 † ‡	90 ± 14 † ‡
	PL	50 ± 7	63 ± 19	82 ± 12† ‡	86 ± 12 † ‡	82 ± 8 † ‡	90 ± 6 † ‡
MAP (mmHg)	PS	73 ± 13	94 ± 12	93 ± 10 † ‡	100 ± 12 † ‡	88 ± 7 † ‡	94 ± 6 † ‡
	PL	70 ± 2	81 ± 17	91 ± 7 † ‡	99 ± 10 † ‡	85 ± 11 † ‡	95 ± 8 † ‡
*f*_R_ (breaths/min)	PS	16 ± 5	19 ± 3	15 ± 5	15 ± 4	19 ± 4	25 ± 9
	PL	18 ± 3	16 ± 7	13 ± 5	13 ± 5	11 ± 4 * ‡	11 ± 4 * ‡
RT (°C)	PS	37.5 ± 0.5	37.0 ± 0.2	36.7 ± 0.3	36.9 ± 0.6	36.7 ± 0.8	36.7 ± 1.0
	PL	37.7 ± 0.8	37.3 ± 0.9	37.2 ± 0.9	37.2 ± 1.0	37.2 ± 1.0	37.2 ± 1.1
SPO_2_	PS	96 ± 2	99 ± 1	99 ± 1	98 ± 2	99 ± 1	97 ± 2
	PL	95 ± 2	100 ± 0	99 ± 1	99 ± 1	97 ± 2	97 ± 2

12 dogs received general anaesthesia with propofol and either epidural application of normal saline (PS) or lidocaine (PL) undergoing ovariohysterectomy.

* Significantly different from other group,

^†^ Significantly different from baseline in the PS group,

^‡^ Significantly different from baseline in PL group.

**Fig 1:**
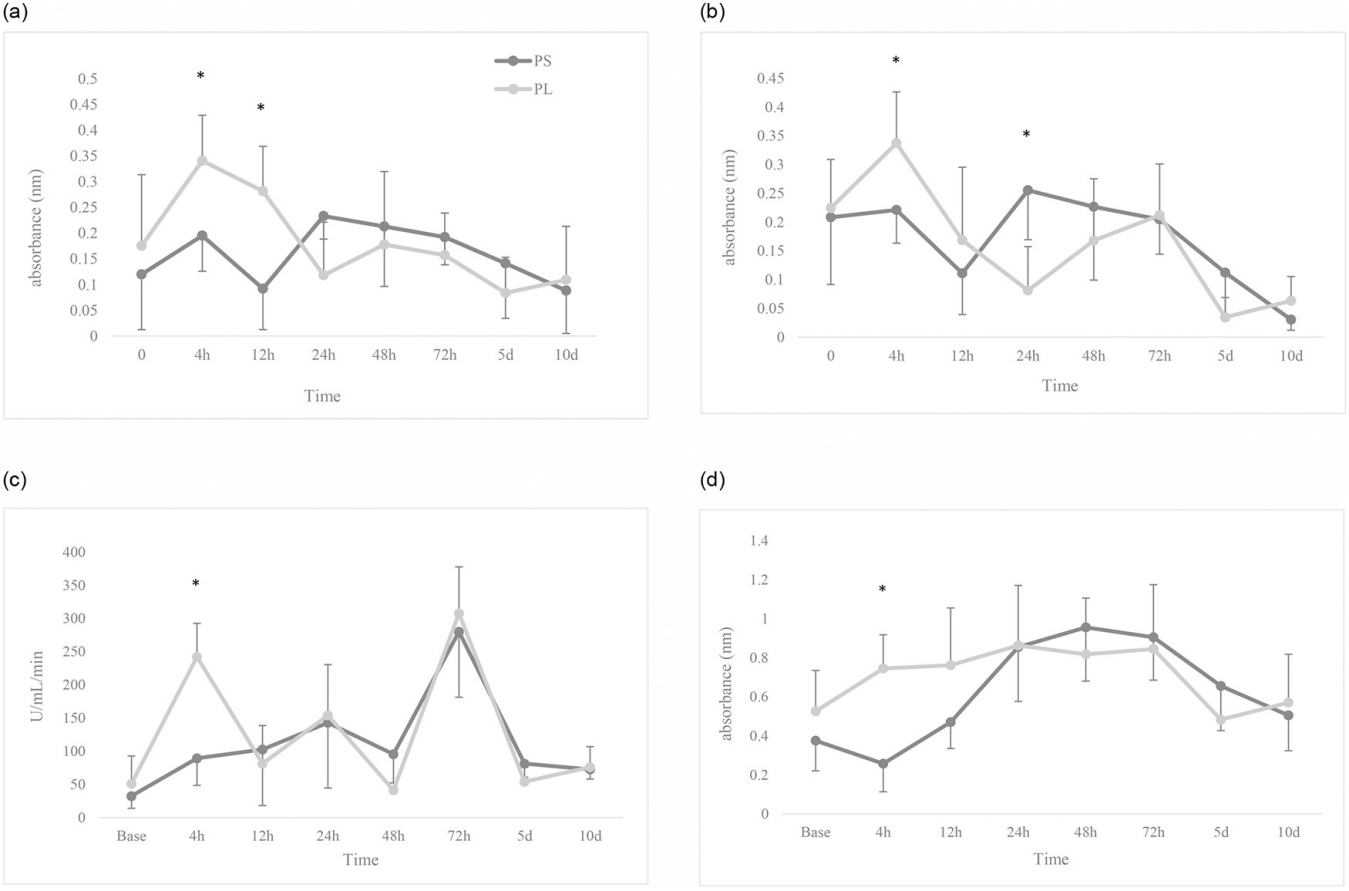
Changes in serum bactericidal activity against *Escherichia coli* (A) and *Staphylococcus aureus* (B), lysozyme (C) and myeloperoxidase (D) activities. 12 dogs received general anaesthesia with propofol and either epidural application of normal saline (PS) or lidocaine (PL) undergoing ovariohysterectomy. Base: immediately after the induction. * Significant difference from other group (*p* < 0.05).

Evaluation of serum bactericidal activity against *Escherichia coli* showed a significant increase at 4 and 12 hours after anaesthesia in PL group compared to PS (*p* < 0.05; Fig 1). Also, bactericidal activity against *Staphylococcus aureus* showed a significant increase at 4 hours and a significant decrease was observed at 24 hours after anaesthesia in PL compared to PS (*p* < 0.05; Fig 1). Evaluation of lysozyme and myeloperoxidase activities showed a significant increase at 4 hours after anaesthesia in the PL group compared to PS (*p* = 0.036 and 0.049, respectively; Fig 1).

**Fig 2:**
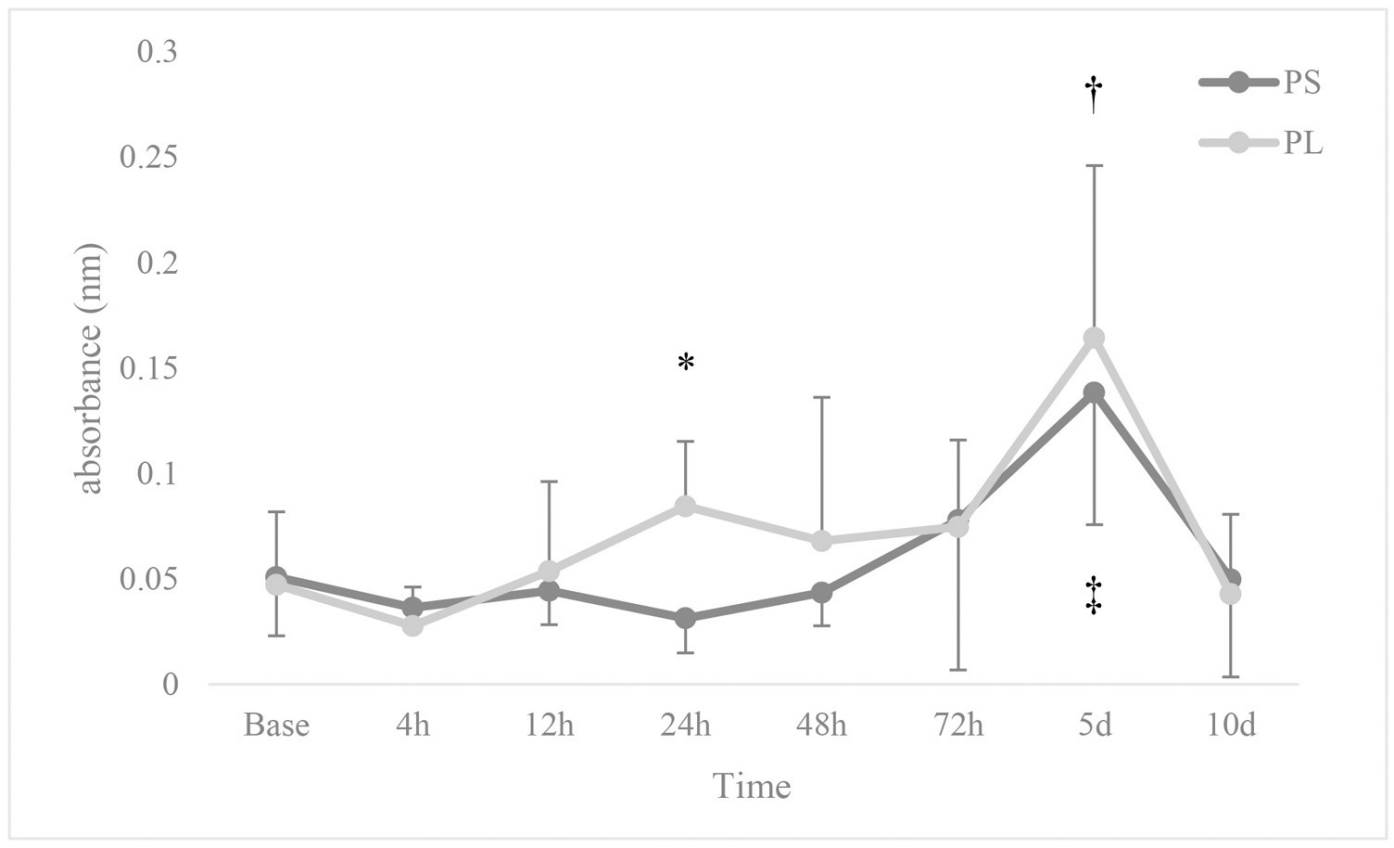
Changes in total immunoglobulin of serum. 12 dogs received general anaesthesia with propofol and either epidural application of normal saline (PS) or lidocaine (PL) undergoing ovariohysterectomy. Base: immediately after induction. * Significantly different from other group, † Significantly different from the baseline in the PS group, ‡ Significantly different from the baseline in PL group.

The total serum immunoglobulin level was higher at 24 hours after induction of anaesthesia in PL compared to that of PS (*p* = 0.049; Fig 2). The total serum immunoglobulin level in the PS and PL groups was significantly higher at 5 days after anaesthesia compared to baseline *p* = 0.048 and 0.045, respectively). There were no significant differences in the levels of anti- SRBC, anti-BSA and anti- *Salmonella typhimurium* antibodies between the two groups and also within each group (*p* > 0.05; Table 2).

**Table 2:** Titers of antibodies against SRBC, BSA and *Salmonella typhimurium*.

	Groups	Base	5 days	10 days
anti-SRBC	PS	0 (0–0)	48 (24–128)	20 (0–128)
	PL	0 (0–8)	56 (4–128)	44 (0–128)
anti-BSA	PS	20 (15–30)	20 (15–40)	40 (0–60)
	PL	35 (20–40)	40 (20–80)	60 (0–60)
anti- *Salmonella typhimurium*	PS	1 (0–2)	8 (2–96)	16 (0–48)
	PL	1 (0–4)	20 (2–96)	20 (0–48)

12 dogs received general anaesthesia with propofol and either epidural application of normal saline (PS) or lidocaine (PL) undergoing ovariohysterectomy.

**Fig 3:**
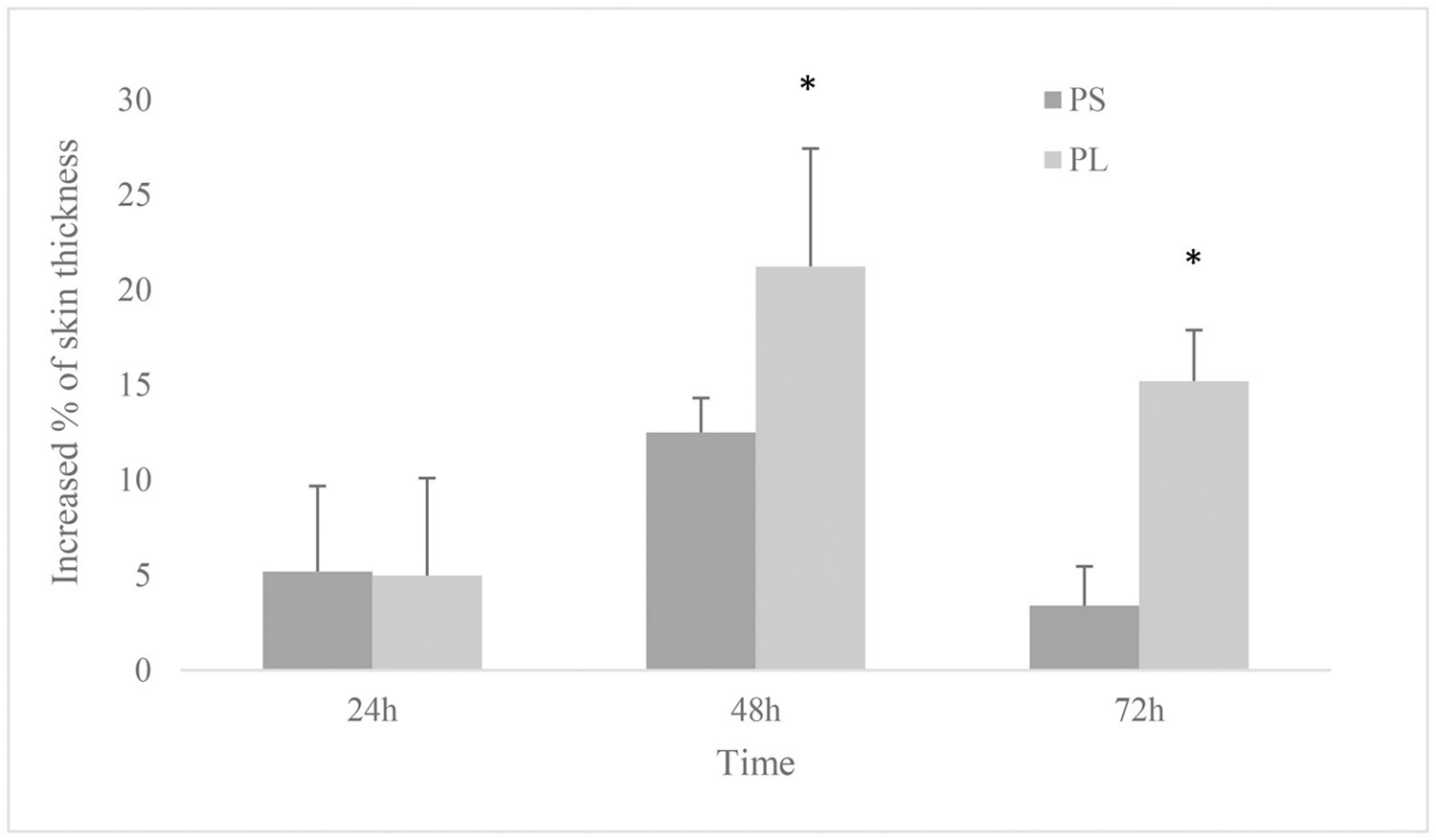
Changes in delayed skin sensitivity to intradermal phytohemagglutinin injection. 12 dogs received general anaesthesia with propofol and either epidural application of normal saline (PS) or lidocaine (PL) undergoing ovariohysterectomy. * Significantly different from the other group.

Evaluation of the delayed skin sensitivity to intradermal phytohemagglutinin injection showed a significant increase at 48 (*p* = 0.045) and 72 (*p* = 0.037) hours after anaesthesia in PL group compared to PS. Assessment of this variable showed no significant changes over time in the two groups compared to the baseline (*p* > 0.05; Fig 3).

**Fig 4:**
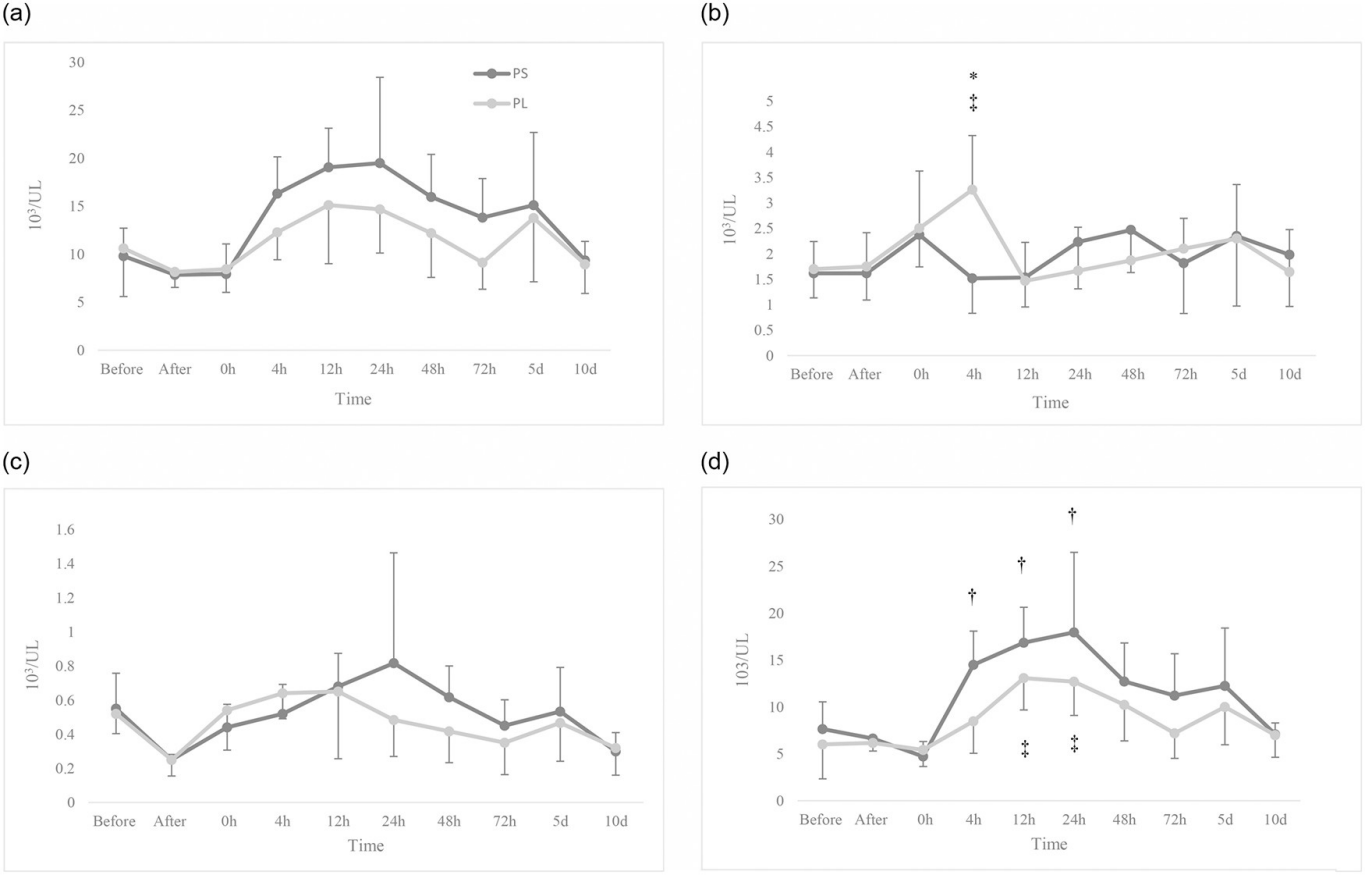
Changes in WBC (a), lymphocytes (b), monocytes (c), and granulocytes counts. 12 dogs received general anaesthesia with propofol and either epidural application of normal saline (PS) or lidocaine (PL) undergoing ovariohysterectomy. Before: before antigen administration; After: after antigen and before sedation administrations. * Significantly different from the other group, † Significantly different from the baseline in the PS group, ‡ Significantly different from the baseline in PL group (*p* < 0.05).

The comparison of WBC count between and within the two groups indicated no significant difference over time (*p* > 0.05). The count of lymphocytes in PL was significantly higher than that of PS at 4 hours after the induction of anaesthesia (*p* = 0.007). Also, at 4 hours after the induction, the amount of lymphocytes was higher in PL group than before the antigen injection (*p* = 0.016). The count of monocytes significantly decreased after the antigen injection compared to before it in the both groups (*p* < 0.05). It was no significant difference with respect to the count of monocytes between the two groups (*p* > 0.05). In the PS group, the count of granulocytes at 4, 12, and 24 hours after the induction was significantly higher than the baseline, while in the PL group, it was higher than the baseline at 12 and 24 hours (*p* < 0.05). The count of granulocytes showed no significant difference between the groups (*p* > 0.05; Fig 4).

**Fig 5:**
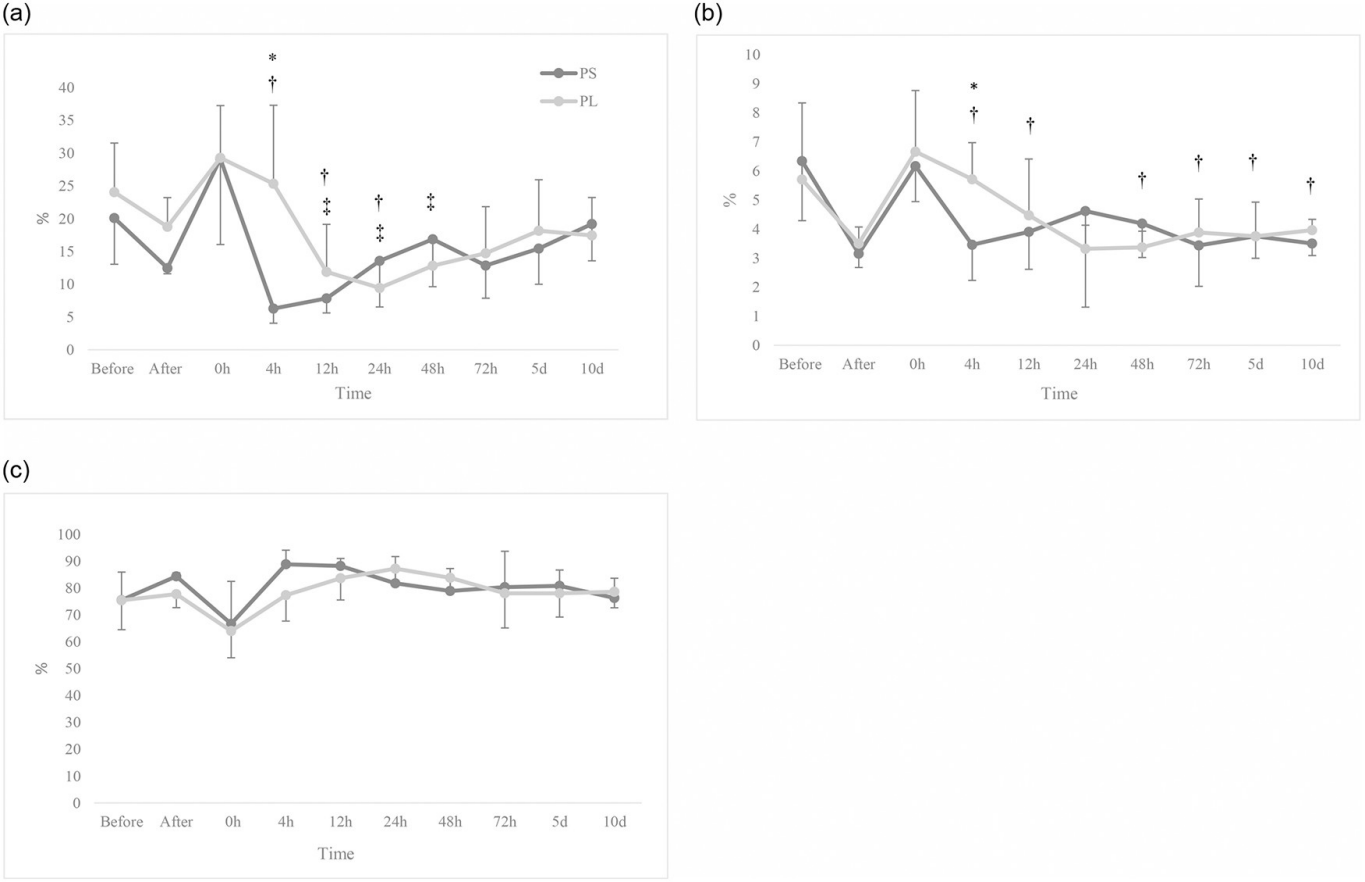
Changes in the percentage of lymphocytes (a), monocytes (b) and granulocytes (c). 12 dogs received general anaesthesia with propofol and either epidural application of normal saline (PS) or lidocaine (PL) undergoing ovariohysterectomy. Before: before antigen administration; After: after antigen and before sedation administrations. * Significantly different from the other group, † Significantly different from the baseline in the PS group, ‡ Significantly different from the baseline in PL group (*p* < 0.05).

The percentage of lymphocytes in the PL group was significantly higher than the PS group at 4 hours after induction (*p* = 0.003). Significant decreases were observed in the percentage of lymphocytes at 4, 12, and 24 hours after anaesthesia in PL and at 12, 24 and 48 hours in PS compared to the before antigen injection (*p* < 0.05). The percentage of monocytes was significantly higher at 4 hours after anaesthesia in the PL group compared to the PS group (*p* = 0.019). The percentage of monocytes in the PS group at 4, 12, 48 and 72 hours, and at 5 and 10 days after induction was significantly lower than before the antigens injection (*p* < 0.05). In PL group, there was no significant difference in the percentage of monocytes over time (*p* > 0.05). Besides, no significant difference were observed in the percentage of granulocytes between and within the two groups (*p* > 0.05; Fig 5).

**Fig 6:**
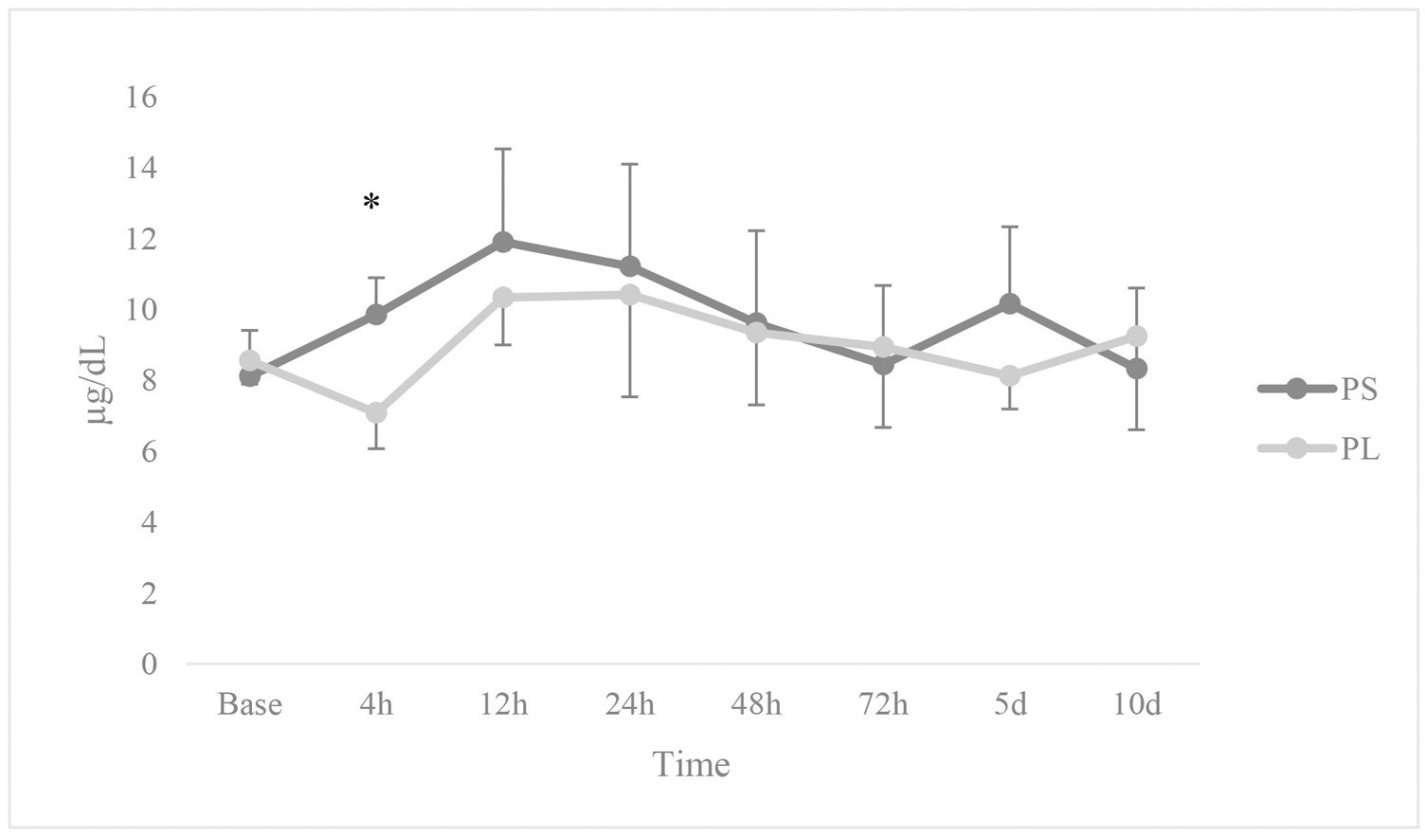
Changes in cortisol levels. 12 dogs received general anaesthesia with propofol and either epidural application of normal saline (PS) or lidocaine (PL) undergoing ovariohysterectomy. * Significantly different from the other group (*p* < 0.05).

Cortisol hormone showed a higher level in PS group than PL at 4 hours after anaesthesia (*p* = 0.024). Comparisons of cortisol hormone did not show a significant change in both groups compared to baseline (*p* > 0.05; Fig 6).

## Discussion

The addition of epidural anaesthesia with lidocaine to general anaesthesia provided by TIVA with propofol in the dogs undergoing OHE operation improved the innate and cellular immune responses. The total immunoglobulin level was higher in the group of dogs that epidurally received lidocaine: however, the other testes for humoral immunity showed no significant alterations. The level of cortisol hormone in the early period of surgery was lesser after lidocaine epidural anaesthesia compared to that of the dogs given the epidural application of normal saline.

The innate immune system was known as responsible for the first responses to the pathogens and injury and comprised of barriers, cytokines, complement, phagocyte and NK cells [27]. In the current investigation, for the innate immune evaluation, bactericidal, lysozyme, and myeloperoxidase functions were assessed. Significant differences were observed in the bactericidal, lysozyme and myeloperoxidase activities at 4 hours after surgery in the dogs receiving epidural lidocaine. When comparison was performed in both of the groups over time, it appears that the innate immunity has been stimulated earlier and responded more intense in group PL compared to PS. Correspondingly, these results indicate that epidural injection of lidocaine inhibited or attenuated the innate immune responses to be suppressed as the same level as those in the dogs receiving no epidural lidocaine. Several human investigations have also showed that innate immune responses have improved after the combination of general anaesthesia and neuraxial blocks [15,19,28]. However, Bar-Yusef et al. [14] found that the number and activity of NK cells have decreased by the addition of spinal block to general anaesthesia in rats; however, pulmonary metastasis has also reduced.

The humoral immunity includes the identification of the antigens and secretion of antibodies by B lymphocytes [27]. Higher values of total immunoglobulin were observed in PL. The level of total immunoglobulin slightly increased in both the groups, reached the highest values at 5 days and then returned to the baseline at 10 days following the surgery. In contrast, although the levels of antibodies against salmonella, BSA and SRBC were higher in PL than those of PS, no significant differences were observed between and within the two treatments. There is a paucity of information with respect to the net effect of anesthetic methods (e.g. general or local anaesthesia) on humoral immune activity. A study in human patients showed that IgM, IgG and IgA were lower than baseline at 30 minutes and 24 hours post anaesthesia with propofol [29]. Propofol has suppressed Th1 and Th2, which stimulates cell-mediated immunity and B lymphocytes to excrete antibodies, respectively [30]; with the same degree in an *in vitro* study [9]. Wada et al. [19] reported that the addition of spinal block to general anaesthesia resulted in Th2 to be dominant over Th1 which might play a role on the progression of tumor metastasis.

In the cellular immune response, a significant increase was observed in PL compared to PS at 48 and 72 hours after the intradermal injection of phytohemagglutinin. In general anaesthesia with either isoflurane or propofol, Th1, as an indicator of cellular immunity, showed lower values [9]. However, the cell-mediated immune impairment has been reported to be lesser in propofol anaesthesia compared to sevoflurane [31,32]. Deegan et al. [33] who measured a number of cytokines involved in cell-mediated immunity, have stated that, the combination of general and paravertebral anaesthesia might be superior to general anaesthesia on the changes of some perioperative cytokines, which might also improve the cellular immunity in women underwent breast cancer surgery.

In hematologic assessment, the number and percentage of lymphocytes and the percentage of monocytes were higher in the group receiving epidural lidocaine compared to the group receiving saline at 4 hours of anaesthesia. Leukocytosis and lymphocytopenia have been reported after propofol anaesthesia in dogs and humans [31,34]. Tomihari et al. [10] reported a reduction in the number of WBC and lymphocytes at 2 first hours following anaesthesia with either propofol or isoflurane in dogs. Nevertheless, they attributed these results to the crystalloid administration and a probable hypotension during the anaesthesia session. Propofol has been proposed that attenuates activation and proliferation of lymphocytes via blocking the potassium channels [35]. Also, epidural anaesthesia has blocked lymphocyte depression produced by general anaesthesia in humans undergoing the orthopedic operation [36]. A study in humans has shown that, the combination of neuraxial block and general anaesthesia decreased the leukocytosis in patients with cancer [33].

Heart rate, MAP and cortisol levels have been employed as the pain indicators in dogs; however, none of them is specific [37]. Although, the changes in HR and MAP were not significant overtime and between the two treatments, the level of cortisol was higher at 4 hours following the anaesthesia in PS than those of PL. The increased production of cortisol is a response to the stress-induced procedures including surgery and anaesthesia, which results in distinct immunosuppression [38]. It has been shown that, the increased concentrations of cortisol are associated with suppression of innate [39] and cellular [40] immune systems, the decreased lymphocyte numbers [41] and changes in the distribution of leukocytes [42]. Several studies have corroborated that neuraxial block when used in combination with general anaesthesia inhibits the cortisol response to surgery due to providing more prominent analgesia [37,43]. Blocking both the afferent and efferent nociceptive pathways has been described as the cause of very effective analgesia produced by epidural anaesthesia [44].

In the current investigation, some immune indices particularly those of innate and cellular immunities showed some degrees of improvement. The first probability is that the addition of epidural anaesthesia to general anaesthesia resulted in a lesser surgery-related stress as well as deeper and longer-lasting analgesia indicated by a lower cortisol level in PL treatment during the first hours following the surgery. It should be noted that to avoid the undesirable consequences of epidural anaesthesia such as hypotension, a relatively low volume of lidocaine (i.e. 0.22 mL/kg) was employed. Although a study on dogs showed that this volume provides acceptable analgesia during OHE operation [45], by considering the low volume and short duration of lidocaine, the effectiveness of the employed protocol for postoperative analgesia is questionable. Therefore, some possibilities such as the systemic analgesia after lidocaine absorption from epidural space and the other characteristics of lidocaine rather than local anaesthesia like anti-inflammatory properties might have been played a role and should be considered [46].

The current study has several limitations. First, a small numbers of dogs were employed which limits the data to be transferrable to larger populations. Although, the sample size was determined to be adequate to show significant differences for repeated measure analyses, for achieving a power of 85% in t-tests, it would need to include at least (optimum effect size of 1) 19 dogs in each group, which was not possible for us. Second, healthy dogs with normal immune functions were selected for the current experiment that is different from clinical situations in which the patients have generally compromised immune system and might differently respond to anaesthesia, surgical stress and pain. Third, because of a grant limitation, we were not able to perform more specific tests focusing on the functions of immune system more precisely to provide more detailed information. Finally, since the impact of some drugs such as opioids have been corroborated on immune system and, in the current investigation, various drugs for premedication, analgesia and antibiotic therapy were used, so the results must be interpreted considering the employed protocol for OHE in the dogs.

In conclusion, epidural administration of lidocaine when added to general anaesthesia with propofol improved the results of the employed tests of immune functions in the dogs undergoing OHE. The protective effect of epidural block was dominant on innate and cellular immunity in the first 4 hours of surgery. Also, a more deep analgesia indicated by a decreased level of cortisol is probably the main factor ror attenuating immune disturbances induced by surgery and anaesthesia. It should be bear in mind that the clinical significance of the obtained results are not clear and caution should be taken in extrapolating the results which must be confirmed by studies with large samples and in clinical situations.

## Supporting information

S1 File Data used for statistical analyses and providing figures and tables. (XLSX)
